# Supplementary material for: Investigation on Pollution Control Device (PCD) in iron foundry industry to reduce environmental chemicals
Source: PLoS One. 2022 Jul 25;17(7):e0271032. doi: 10.1371/journal.pone.0271032 (PMC9312380; doi:10.1371/journal.pone.0271032)
Supplement: S1 File — (DOCX) [file pone.0271032.s001.docx]

**Investigation on Pollution Control Device (PCD) in Iron Foundry Industry to Reduce Environmental Chemicals**

**Krishnaraj Ramaswamy^1,3,*^, Leta Tesfaye Jule^2,3^, Nagaprasad. N^4^, Kumaran Subramanian^5^, Shanmugam. R^6^, Priyanka Dwarampudi. L^7^, Venkatesh Seenivasan^8^**

^1^Dambi Dollo University, College of Engineering Science, Mechanical Engineering Department, Ethiopia.

^2^Dambi Dollo University, College of Natural and Computational Science, Department of Physics, Ethiopia.

^3^Centre for Excellence in Technology Transfer and Incubation, Dambi Dollo University, Ethiopia.

^4^Department of Mechanical Engineering, ULTRA College of Engineering and Technology, Madurai – 625 107, Tamil Nadu, India.

^5^Centre for Drug Discovery and development, Sathyabama Institue of science and technology, Chennai-600119, Tamilnadu, India.

^6^TIFAC, CORE-HD, Department of Pharmacognosy, JSS College of Pharmacy, JSS Academy of Higher Education & Research, Ooty, Nilgiris, Tamil Nadu, India.

^7^Department of Pharmacognosy, JSS College of Pharmacy, JSS Academy of Higher Education & Research, Ooty, Nilgiris, Tamil Nadu, India.

^8^Department of Mechanical Engineering, Sri Eshwar College of Engineering, Coimbatore, Tamil Nadu, India.

*Corresponding Author

Email: [prof.dr.krishnaraj@dadu.edu.et](mailto:prof.dr.krishnaraj@dadu.edu.et) (RK)

**Supplementary data**

**S1 Table.** Particulate Matter Concentration Emission Review.

| **Sl.No** | **Pollution Control Device** | **Particulate Matter concentration (mg/Nm^3^)** |
| --- | --- | --- |
| 1 | Cartridge filter | 20 |
| 2 | Wet scrubber | 38 |
| 3 | Cyclones | 50 |
| 4 | Venturi wet scrubber | 67.90 |
| 5 | Wetcap collectoer | 70 |
| 6 | Wetcap collector | 70 |
| 7 | Drycap collector | 107.90 |
| 8 | Dry cap collector | 350 |

**S2 Table.** PCDs Installed in Foundries of Tamilnadu State and Their Impact on Pollution Prevention Stack temperature (average) in degree Kelvin

| **Iron**  **Foundry**  **number** | **Furnace**  **used** | **PCD** | **Stack temperature**  **(average) in degree Kelvin** | |
| --- | --- | --- | --- | --- |
|  |  |  | **Before** | **After** |
|  |  |  | **Installing the PCD** | |
| 1 | Cupola | Cyclone | 200 | 39 |
| 2 | Cupola | Wet cap  Collector | 200 | 150 |
| 3 | Cupola | Dry cap  collector | 250 | 200 |
| 4 | Cupola | Wet cap  collector | - | 150 |
| 5 | Cupola | Venturi wet  Scrubber | - | 37 |
| 6 | Cupola | Wet cap  Collector | - | 150 |
| 7 | Cupola | Drycap collector | - | 200 |
| 8 | Cupola | Wet cap  Collector | - | 150 |
| 9 | Cupola | Drycap collector | - | 200 |
| 10 | Cupola | Wet cap  collector | - | 160 |
| 11 | Induction | Venturi wet scrubber | - | 28 |
| 12 | Induction | Venturi wet  scrubber | - | 29 |
| 13 | Induction | Wet scrubber | - | 53 |
| 14 | Induction | Wet scrubber | - | 38 |
| 15 | Induction | Wet scrubber | - | 32 |
| 16 | Induction | Wet scrubber | - | 35 |
| 17 | Induction | Wet scrubber | - | 26 |
| 18 | Induction | Wet scrubber | - | 36 |
| 19 | Induction | Wet scrubber | - | 33 |
| 20 | Induction | Wet scrubber | - | 28 |
| 21 | Induction | Cartridge filter | 200 | <50 |

**S3 Table.** PCDs Installed in Foundries of Tamilnadu State: Discharge rate

(liters/hour)

| **Iron**  **Foundry**  **number** | **Furnace**  **used** | **PCD** | **Velocity**  **(m/sec)** | **Discharge rate**  **(liters/hour)** | |
| --- | --- | --- | --- | --- | --- |
|  |  |  |  | **Before** | **After** |
|  |  |  |  | **Installing the PCD** | |
| 1 | Cupola | Cyclone | 7.4 | 8350 | 4099 |
| 2 | Cupola | Wet cap  Collector | - | 5670 | 4500 |
| 3 | Cupola | Dry cap  collector | - | - | - |
| 4 | Cupola | Wet cap  collector | 5.52 | - | 1060 |
| 5 | Cupola | Venturi wet  Scrubber | 11.4 | - | 2043 |
| 6 | Cupola | Wet cap  Collector | 11.28 | - | 1066 |
| 7 | Cupola | Drycap collector | - | - | - |
| 8 | Cupola | Wet cap  Collector | 11.4 | - | 1757 |
| 9 | Cupola | Drycap collector | - | - | - |
| 10 | Cupola | Wet cap  collector | 6.2 | - | 1360 |
| 11 | Induction | Venturi wet scrubber | 9.86 | - | 5588 |
| 12 | Induction | Venturi wet  scrubber | <10 | - | 4926 |
| 13 | Induction | Wet scrubber | 9.86 | - | 5588 |
| 14 | Induction | Wet scrubber | 9.2 | - | 5260 |
| 15 | Induction | Wet scrubber | 10.2 | - | 4592 |
| 16 | Induction | Wet scrubber | 11 | - | 6120 |
| 17 | Induction | Wet scrubber | 9.8 | - | 4980 |
| 18 | Induction | Wet scrubber | 10.5 | - | 5950 |
| 19 | Induction | Wet scrubber | 10 | - | 3500 |
| 20 | Induction | Wet scrubber | 9.1 | - | 5120 |
| 21 | Induction | Cartridge filter | 9 | 40000 | 5185 |

**S4 Table.** PCDs Installed in Foundries of Tamilnadu State: The concentration of pollutants in (mg/Nm^3^)

| **Iron**  **Foundry**  **number** | **Furnace**  **used** | **PCD** | **SPM**  **(mg/Nm^3^)**  **(average)** | | **The concentration of pollutants in (mg/Nm^3^)** | | | |
| --- | --- | --- | --- | --- | --- | --- | --- | --- |
|  |  |  | **Before** | **After** | **SO_2_** | **NO_x_** | **CO_2_** | **CO** |
|  |  |  | **Installing the PCD** | |  |  |  |  |
| 1 | Cupola | Cyclone | 300 | 67 | 2.7 | 1.2 | - | - |
| 2 | Cupola | Wet cap  Collector | 2500 | 110 | 10 | 17 | - | 0.2 |
| 3 | Cupola | Dry cap  collector | 1750 | 496 | 340 | 18 | 4.5 | - |
| 4 | Cupola | Wet cap  collector | - | 110 | 31 | 21 | - | 0.2 |
| 5 | Cupola | Venturi wet  Scrubber | - | 51 | 26 | 11 | - | 0.2 |
| 6 | Cupola | Wet cap  Collector | - | 102 | 21 | 15 | - | - |
| 7 | Cupola | Drycap collector | - | 394 | 38 | 18 | - | - |
| 8 | Cupola | Wet cap  Collector | - | 95 | 66 | 20 | 5 | - |
| 9 | Cupola | Drycap collector | - | 366 | 63 | 47 | ND | - |
| 10 | Cupola | Wet cap  collector | - | 105 | 30 | 19 | ND | - |
| 11 | Induction | Venturi wet scrubber | - | 31 | 19.2 | 3.1 | 5 | ND |
| 12 | Induction | Venturi wet  scrubber | - | 37 | 21 | 12 | ND | ND |
| 13 | Induction | Wet scrubber | - | 52 | 19.2 | 31 | ND | 0.2 |
| 14 | Induction | Wet scrubber | - | 51 | 12 | 7 | ND | ND |
| 15 | Induction | Wet scrubber | - | 50 | 15 | 8 | ND | ND |
| 16 | Induction | Wet scrubber | - | 51 | 12 | 6 | ND | ND |
| 17 | Induction | Wet scrubber | - | 49 | 14 | 7 | ND | ND |
| 18 | Induction | Wet scrubber | - | 55 | 10 | 5 | ND | ND |
| 19 | Induction | Wet scrubber | - | 50 | 21 | 11 | ND | ND |
| 20 | Induction | Wet scrubber | - | 48 | 18 | 10 | ND | ND |
| 21 | Induction | Cartridge filter |  | <20 | ND | ND | ND | ND |
