# Supplementary material for: Investigation on Pollution Control Device (PCD) in iron foundry industry to reduce environmental chemicals
Source: PLoS One. 2022 Jul 25;17(7):e0271032. doi: 10.1371/journal.pone.0271032 (PMC9312380; doi:10.1371/journal.pone.0271032)
Supplement: Attachment — Submitted filename: Comments.docx [file pone.0271032.s002.docx]

**Investigation on Pollution Control Device (PCD) in Foundry Industry to Reduce Environmental Chemicals**

The manuscript titled “Investigation on Pollution Control Device (PCD) in Foundry Industry to Reduce Environmental Chemicals” written by authors is a good informative work in the field and useful for future researchers. The article ends by emphasizing the need to conduct surveys in foundries in which a cartridge filter is installed. The results of this study will provide useful information on the use of cartridge filters in induction furnaces to reduce foundry emissions. Though the results seem to be convincing there are some issues to be resolved in this manuscript. Addressing the below comments, I recommend the article to be published in the journal.

Recommendation: **Major Revision**

**Comments:**

1. In the abstract section, include the result of cartridge filter equipped with an Induction furnace.
2. In the result and discussion section compare the application of low energy scrubbers and High scrubbers from literature.
3. Check the units throughout the manuscript.
4. Since the authors has concentrated in Iron Foundry Industry. Hence the title has to revise as “Investigation on Pollution Control Device (PCD) in Iron Foundry Industry to Reduce Environmental chemicals.
5. What are the other industries which use the pollution control devices?
6. In general the manuscript needs editing in terms of language and choice of words for example in some places the word smelters was used. Instead of foundries. The clarity of the manuscript should be improved.
