# Supplementary material for: Investigation on Pollution Control Device (PCD) in iron foundry industry to reduce environmental chemicals
Source: PLoS One. 2022 Jul 25;17(7):e0271032. doi: 10.1371/journal.pone.0271032 (PMC9312380; doi:10.1371/journal.pone.0271032)
Supplement: Attachment — Submitted filename: Response to review comments Comments.docx [file pone.0271032.s003.docx]

**RESPONSE TO REVIEWERS COMMENTS**

**Paper Title: Investigation on Pollution Control Device (PCD) in Iron Foundry Industry to Reduce Environmental Chemicals**

Dear Editor,

Coauthors and I very much appreciated the encouraging, critical and constructive comments on this manuscript by the reviewer. The comments have been very thorough and useful in improving the manuscript. We strongly believe that the comments and suggestions have increased the scientific value of the revised manuscript by many folds. We have taken them fully into account in the revision. We are submitting the corrected manuscript with the suggestion incorporated into the manuscript (It is highlighted in red colour). The manuscript has been revised as per the comments given by the reviewer, and our responses to all the comments are as follows:

**Reviewer 2:**

**1. In the abstract section, include the result of cartridge filter equipped with an Induction furnace.**

Dear Professor, Thank you, The abstract section has been revised as per your suggestion

**Abstract**

Right from the olden days, many products have been made according to foundry practices in order to generate prosperity in the societies in which they operate, while reaping these types of benefits through the operation of foundries. It is alarming that the emissions released by foundries affect human health. Therefore, foundries installed Pollution Control devices (PCDs), in accordance with this development; researchers examined the effectiveness of these PCDs in controlling emissions from foundries in different parts of the world. The emission control obtained by installing these PCDs is explained in this article based on the data gathered from the survey. The cartridge filter equipped with an induction furnance reduced the concentration of SPM to less than 20 mg/Nm^3^. This result of the investigation indicates that the catridge filter built into the induction furnance achieves the best efficiency in controlling contaminants from iron foundries. Interestingly, the operation of the catridge filter has yet to be documented. Therefore, the construction operation, the performance of the cartridge filter, and its efficiency in achieving contaminations control in foundries are described. Which will provide useful information on the use of cartridge filters in induction furnance to reduce Iron foundry emissions.

**2. In the result and discussion section compare the application of low energy scrubbers and High scrubbers from literature.**

 **Fig. 4.** Energy Scrubber in Iron Foundary Industries.

Dear Professor, Thank you, As per your suggestion the applications are included in the revised version of the manuscript.

**3. Check the units throughout the manuscript.**

Throughout the manuscript the units are verified and it is highlighted in red colour.

**4. Since the authors has concentrated in Iron Foundry Industry. Hence the title has to revise as “Investigation on Pollution Control Device (PCD) in Iron Foundry Industry to Reduce Environmental chemicals.**

As per your suggestion the title is modified.

**Investigation on Pollution Control Device (PCD) in Iron Foundry Industry to Reduce Environmental Chemicals**

**5. What are the other industries which use the pollution control devices?**

The pollution control devices are mostly utilized in foundries to separate the particulate emission from the furnace. Moreover, the pollution control devices are utilized in chemical engineering industries, cement industries, textile industries, mining industries, and paper mills to separate the particulate emission from the air/gas stream. In addition, the pollution control devices are utilized in the circulating fluidized bed (CFB) combustion process to separate the various particulate matters and pollutant gases.

**6. In general the manuscript needs editing in terms of language and choice of words for example in some places the word smelters was used. Instead of foundries. The clarity of the manuscript should be improved**

As per your suggestion, throughout the manuscript, the corrections are included.

**Reviewer 1:**

1. **The manuscript is not research article or a review article, both mixed to together and confuses the reader.**

Dear Professor, Thank you for your valuable comments.

- This is a research article which addresses the research gap through survey, investigation and literature gap.
- The manuscript pointed out the direction of practice and literature gab to bring about prosperity to the societies by running Iron foundries that cause no pollution.
- One of the findings of this research is that the usage of an induction furnace for melting the metals in iron foundries leads to the efficient controlling of pollution.
- Another Finding is the cartridge filter, integrated with induction furnaces, exits only 20 mg/Nm^3^ of pollutants, which will be considered the most economical and efficient pollution control device that can be used for controlling pollution in foundries.

2. **The abstract not mentioning the results obtained from authors work. I feel it is lack of confidence.**

The abstract section has been revised as per your suggestion

**Abstract**

Right from the olden days, many products have been made according to foundry practices in order to generate prosperity in the societies in which they operate, while reaping these types of benefits through the operation of foundries. It is alarming that the emissions released by foundries affect human health. Therefore, foundries installed Pollution Control devices (PCDs), in accordance with this development; researchers examined the effectiveness of these PCDs in controlling emissions from foundries in different parts of the world. The emission control obtained by installing these PCDs is explained in this article based on the data gathered from the survey. The cartridge filter equipped with an induction furnace reduced the concentration of SPM to less than 20 mg/Nm^3^. This result of the investigation indicates that the cartridge filter built into the induction furnace achieves the best efficiency in controlling contaminants from iron foundries. Interestingly, the operation of the cartridge filter has yet to be documented. Therefore, the construction operation, the performance of the cartridge filter, and its efficiency in achieving contaminations control in foundries are described. This will provide useful information on the use of cartridge filters in induction furnace to reduce Iron foundry emissions.

**3. Entire manuscript flow is few para review and another few what to be done but author failed to impress based on the work what they have done experimentally. The cartridge figure is computer design diagram, that create the doubt whether this work is done on the foundry or not. Rewrite the manuscript with proper results and discussion and methods used to conclude the results with references. No error data present.**

Dear Professor, thanks for your suggestions; now, the manuscript quality has been improved.

**Table 1.** Particulate Matter Concentration Emission Review.

| **Sl.No** | **Pollution Control Device** | **Particulate Matter concentration (mg/Nm^3^)** | **Reference** |
| --- | --- | --- | --- |
| 1 | Cartridge filter | 20 | Current Paper |
| 2 | Wet scrubber | 38 | Kumar et al. (2020) |
| 3 | Cyclones | 50 | Guinot et al. (2016) |
| 4 | Venturi wet scrubber | 67.90 | Aksu et al. (2015) |
| 5 | Wetcap collectoer | 70 | Safar et al. (2010) |
| 6 | Wetcap collector | 70 | Pal et al. (2008) |
| 7 | Drycap collector | 107.90 | Pineda-Martinez et al. (2014) |
| 8 | Dry cap collector | 350 | Fatta et al. (2004) |

- Our research result has been validated through the above table.
- Although relatively less number of researches on controlling pollution is reported in the literature arena, the construction and working of cartridge filters are not available in the literature; hence the construction and working of the cartridge filter are discussed so that this contribution will suggest the Foundry engineers in choosing cartridge filter as a pollution control device in the iron foundries, which will facilitate the hassle-free running of iron foundries that will enhance the prosperities of societies in which of these iron foundries are located.
